# Supplementary figures and images for: A Novel Diphenylthiosemicarbazide Is a Potential Insulin Secretagogue for Anti-Diabetic Agent
Source: PLoS One. 2016 Oct 20;11(10):e0164785. doi: 10.1371/journal.pone.0164785 (PMC5072725; doi:10.1371/journal.pone.0164785)

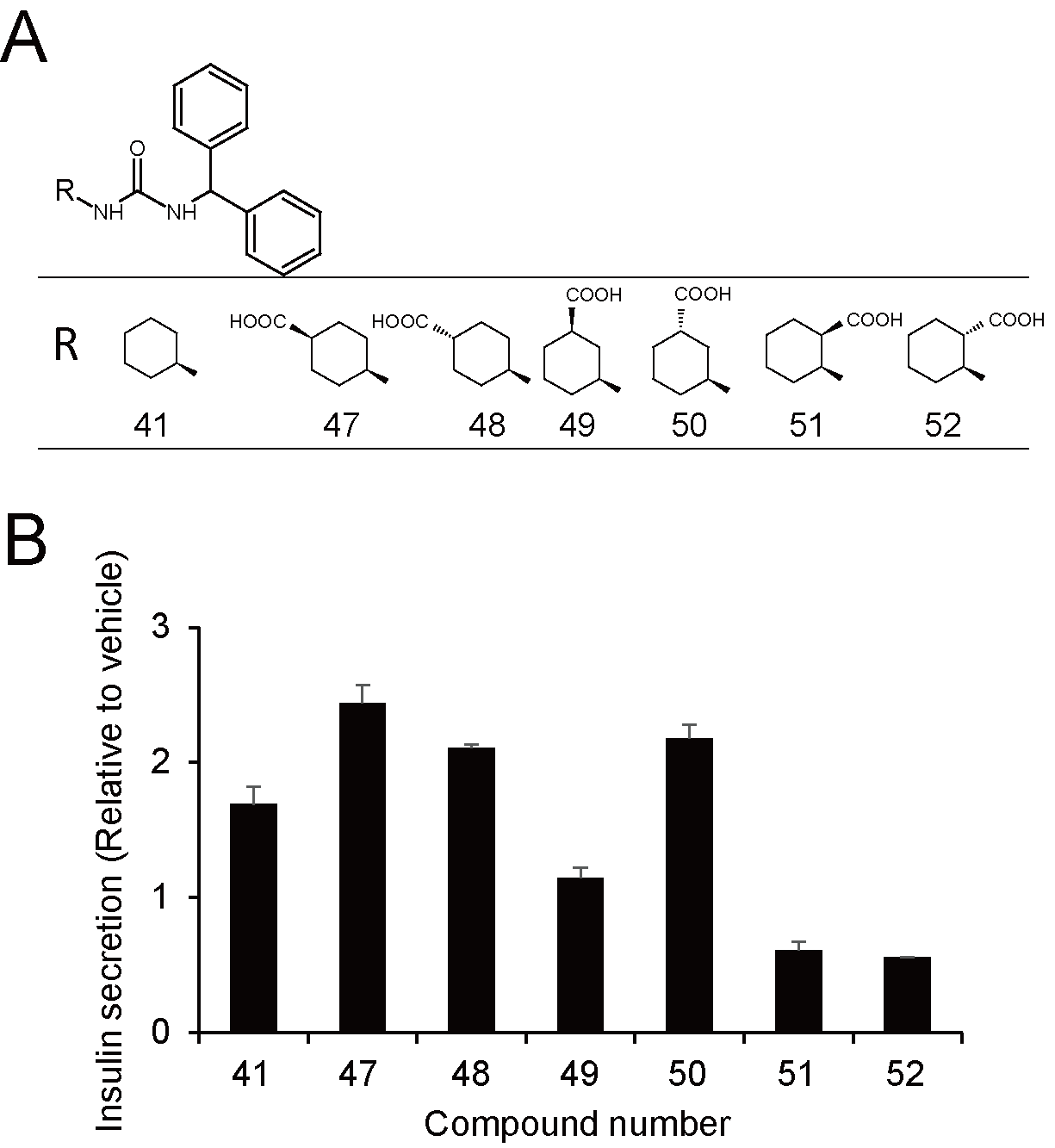

Supplement: S1 Fig — (A) Structures of carboxylated C8-derivatives. Carboxylation to various sites on cyclohexane were introduced to C41, one of the C8-derivatives. (B) Effects of carboxylated derivatives on insulin secretion. MIN6-K8 cells were stimulated by 100 μM of each compound. Data are shown as fold-increase in insulin secretion relative to vehicle. Data are expressed as mean ± SEM (n = 3 for each compound) (TIF) [file pone.0164785.s001.tif]

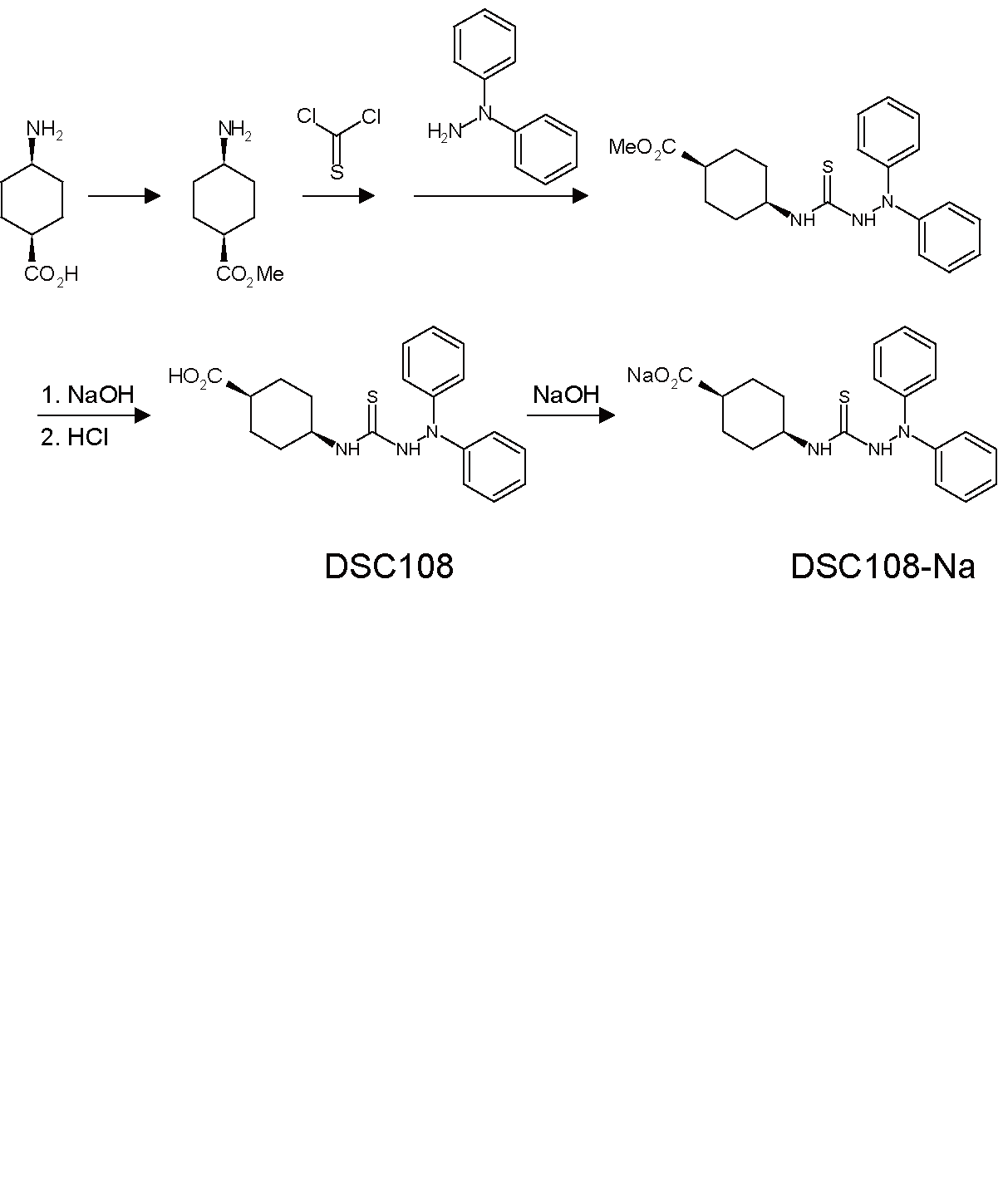

Supplement: S2 Fig — (TIF) [file pone.0164785.s002.tif]

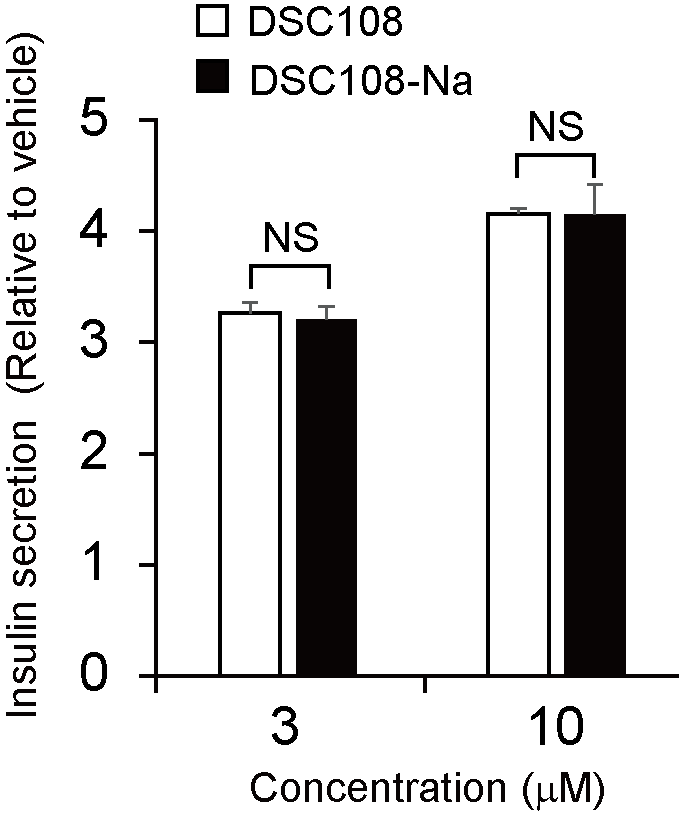

Supplement: S3 Fig — Insulin secretion from MIN6-K8 cells stimulated by 3 or 10 μM of each compound in the presence of 11.2 mM glucose. Data are shown as fold-increase in insulin secretion relative to vehicle. Data are expressed as mean ± SEM (n = 3 for each compound). NS, not significant (Student unpaired t test). (TIF) [file pone.0164785.s003.tif]

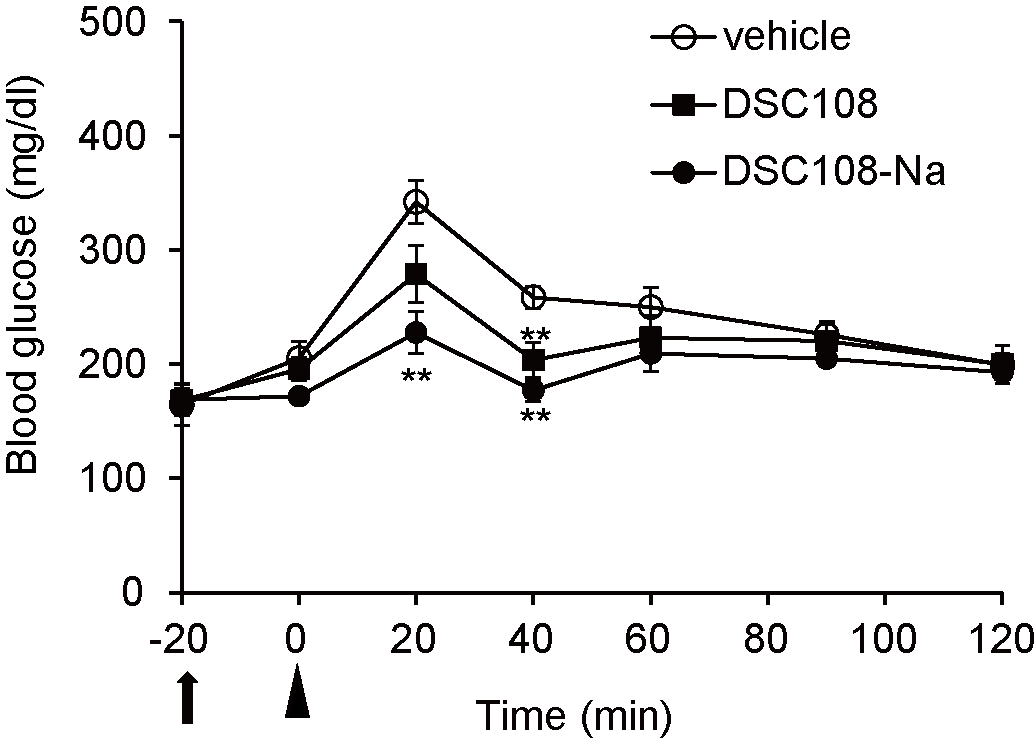

Supplement: S4 Fig — Blood glucose levels after oral glucose load following administration of DSC108 and DSC108-Na were monitored. Vehicle or 30 mg/kg of DSC108 or DSC108-Na were administered orally at -20 min and glucose (1.5g/kg) was administered orally at 0 min. Data are expressed as mean ± SEM (n = 4 for each group). Arrow and arrowhead indicate the administration of compound and glucose, respectively. **P < 0.01 vs. vehicle group (Dunnet’s method) (TIF) [file pone.0164785.s004.tif]

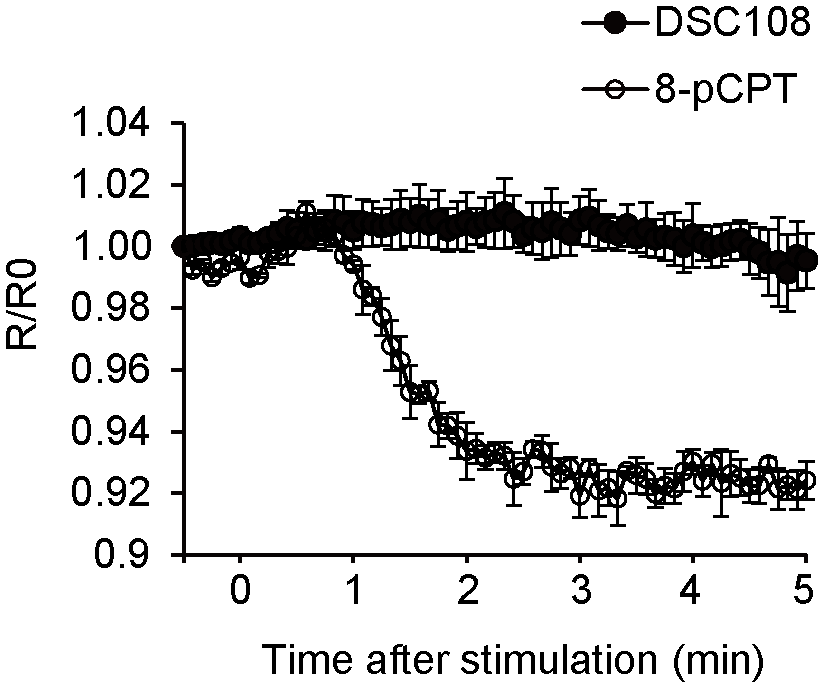

Supplement: S5 Fig — Effect of DSC108 on Epac2A activation in MIN6-K8 cells was assessed by FRET experiment as reported previously [11]. MIN6-K8 cells were transfected with mouse wild-type Epac2A FRET sensor. FRET emission ratio from the cells stimulated by 10 μM of DSC108 or 10 μM 8-pCPT (8-pCPT-2’-O-Me-cAMP, an Epac-selective cAMP analog) was monitored as previously described [12]. (TIF) [file pone.0164785.s005.tif]

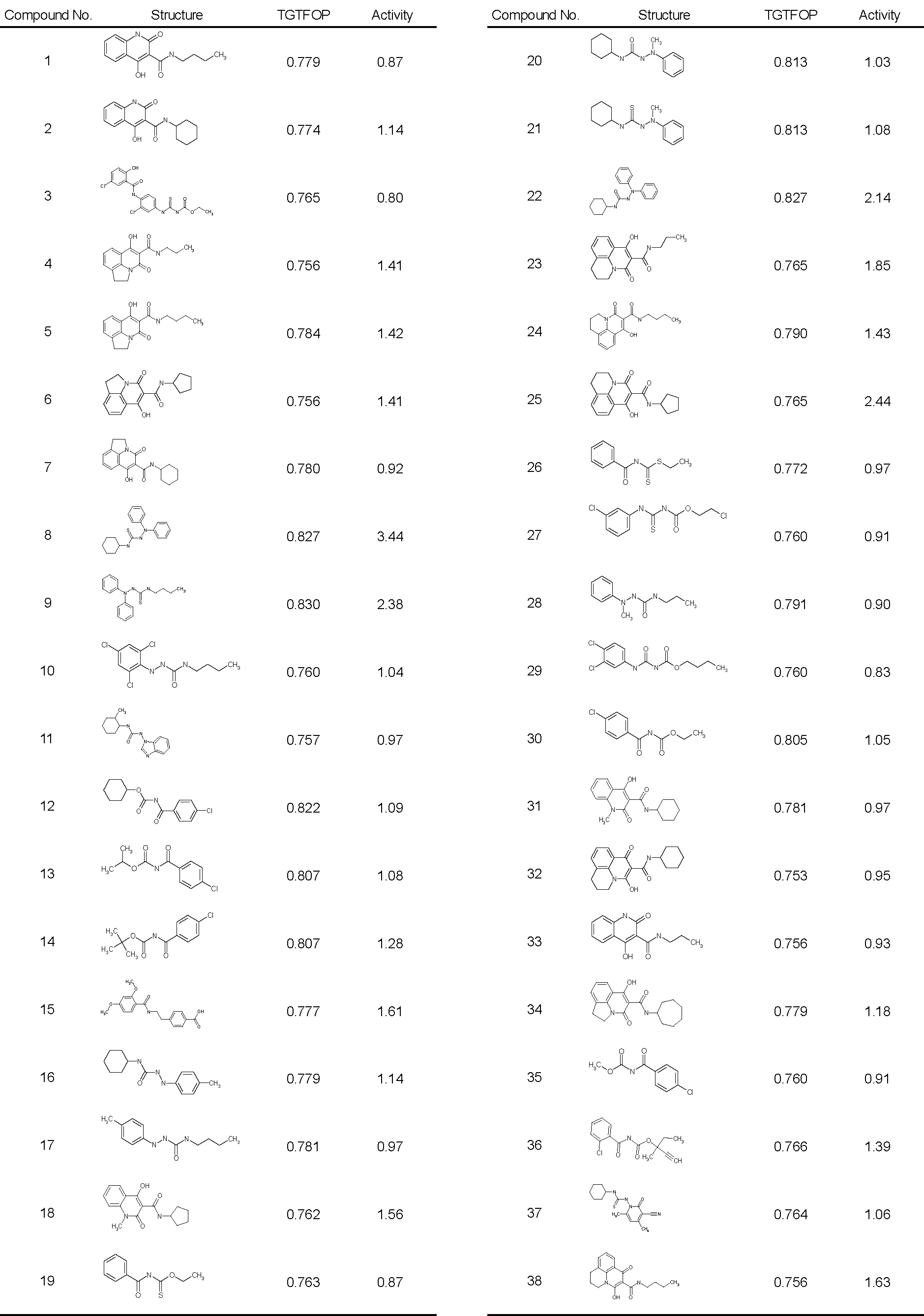

Supplement: S1 Table — Structures, values of TGTFOP calculated by in silico similarity search, and activities obtained by the first screening (Fig 1A) are shown. (TIF) [file pone.0164785.s006.tif]
