# Supplementary material for: Correction: A Novel Diphenylthiosemicarbazide Is a Potential Insulin Secretagogue for Anti-Diabetic Agent
Source: PLoS One. 2017 Jan 12;12(1):e0170430. doi: 10.1371/journal.pone.0170430 (PMC5231920; doi:10.1371/journal.pone.0170430)
Supplement: S2 File — (PDF) [file pone.0170430.s002.pdf]

RESEARCH ARTICLE

A Novel Diphenylthiosemicarbazide Is a Potential Insulin Secretagogue for Anti-Diabetic Agent

Kenji Sugawara[1,2], Kohei Honda[1], Yoshie Reien[3], Norihide Yokoi[1], Chihiro Seki[1], Harumi Takahashi[1], Kohtaro Minami[1], Ichiro Mori[4], Akio Matsumoto[3], Haruaki Nakaya[3], Susumu Seino[1]*

1 Division of Molecular and Metabolic Medicine, Kobe University Graduate School of Medicine, Kobe, Japan, **2** Division of Diabetes and Endocrinology, Kobe University Graduate School of Medicine, Kobe, Japan, **3** Department of Pharmacology, Graduate School of Medicine, Chiba University, Chiba, Japan, **4** Division of Advance Medical Science, Graduate School of Science, Technology and Innovation, Kobe University, Kobe, Japan

* seino@med.kobe-u.ac.jp

OPEN ACCESS

Citation: Sugawara K, Honda K, Reien Y, Yokoi N, Seki C, Takahashi H, et al. (2016) A Novel Diphenylthiosemicarbazide Is a Potential Insulin Secretagogue for Anti-Diabetic Agent. PLoS ONE 11(10): e0164785. doi:10.1371/journal.pone.0164785

Editor: Makoto Kanzaki, Tohoku University, JAPAN

Received: June 30, 2016

Accepted: October 2, 2016

Published: October 20, 2016

Copyright: © 2016 Sugawara et al. This is an open access article distributed under the terms of the Creative Commons Attribution License, which permits unrestricted use, distribution, and reproduction in any medium, provided the original author and source are credited.

Data Availability Statement: All relevant data are within the paper and its Supporting Information files.

Funding: This work was supported by Grant-in-Aid for Scientific Research from the Ministry of Education, Culture, Sports, Science and Technology, Japan. This study was also supported in part by a research grant from MSD K.K. to SS and by a grant for Young Researchers from the Japan Association for Diabetes Education and Care to KS. The funders had no role in study design, data collection and analysis, decision to publish, or preparation of the manuscript.

Abstract

Insulin secretagogues are used for treatment of type 2 diabetes. We attempted to discover novel small molecules to stimulate insulin secretion by using in silico similarity search using sulfonylureas as query, followed by measurement of insulin secretion. Among 38 compounds selected by in silico similarity search, we found three diphenylsemicarbazides and one quinolone that stimulate insulin secretion. We focused on compound 8 (C8), which had the strongest insulin-secreting effect. Based on the structure-activity relationship of C8-derivatives, we identified diphenylthiosemicarbazide (DSC) 108 as the most potent secretagogue. DSC108 increased the intracellular Ca^{2+} level in MIN6-K8 cells. Competitive inhibition experiment and electrophysiological analysis revealed sulfonylurea receptor 1 (SUR1) to be the target of DSC108 and that this diphenylthiosemicarbazide directly inhibits ATP-sensitive K^+ (K_{ATP}) channels. Pharmacokinetic analysis showed that DSC108 has a short half-life in vivo. Oral administration of DSC108 significantly suppressed the rises in blood glucose levels after glucose load in wild-type mice and improved glucose tolerance in the Goto-Kakizaki (GK) rat, a model of type 2 diabetes with impaired insulin secretion. Our data indicate that DSC108 is a novel insulin secretagogue, and is a lead compound for development of a new anti-diabetic agent.

Introduction

Type 2 diabetes is characterized by impaired insulin secretion from pancreatic β-cells and impaired insulin sensitivity in target tissues including liver, adipose tissues, and muscles. Various anti-diabetic drugs to stimulate insulin secretion from pancreatic β-cells and to improve insulin sensitivity in insulin target tissues have been developed to date. Insulin secretagogues including sulfonylureas, glinides, and incretin-related drugs are widely used in clinical practice

Competing Interests: The authors have declared that no competing interests exist.

for treatment of patients with type 2 diabetes with impaired insulin secretion [1]. Among these, sulfonylureas are the most commonly used anti-diabetic drug worldwide. Sulfonylureas inhibit the ATP-sensitive K^+ (K_{ATP}) channels in the pancreatic β-cells to simulate insulin secretion [2–5]. The β-cell K_{ATP} channel is composed of Kir6.2, the pore-forming subunit, and SUR1, a sulfonylurea receptor, as the regulatory subunit [6–8]. Sulfonylureas and glinides bind to SUR1 to induce closure of the K_{ATP} channels, depolarizing the β-cell membrane, leading to opening of the voltage-dependent Ca^{2+} channels (VDCCs), and allowing Ca^{2+} influx into the β-cells. The resultant rise in intracellular Ca^{2+} concentration ($[Ca^{2+}]i$) triggers insulin release [8]. Thus, in addition to its physiologically essential role as an ATP sensor in glucose-induced insulin secretion (GIIS), the K_{ATP} channel is a validated drug target to regulate insulin secretion [2–5, 9]. However, despite their beneficial effects, these drugs can cause prolonged hypoglycemia, especially for elderly patients and patients with renal insufficiency, due in part to their long half-life properties [10].

We recently reported that in addition to causing closure of the K_{ATP} channels, sulfonylureas directly activate Epac2A/Rap1 signaling in pancreatic β-cells to stimulate insulin secretion [11, 12]. We also have found that sulfonylureas and cAMP cooperatively activate Epac2A to simulate insulin secretion [13]. Sulfonylureas are reported to act on sulfonylurea receptor-like molecules on insulin granules [14–16]. Thus, sulfonylureas and sulfonylureas-related molecules have diverse targets in the insulin secretory mechanism [17].

In silico screening is widely used as a powerful and effective approach for the discovery of novel therapeutic compounds. Similarity search is an especially useful method for retrieving compounds with characteristics similar to those of known ligands [18]. In the present study, we attempted to identify novel compounds to stimulate insulin secretion by similarity search utilizing the information on the structures of the sulfonylureas in combination with measurement of their insulinotropic effects both in vitro and in vivo. We identified a diphenylthiosemicarbazide-derivative (designated DSC108) as a novel insulin secretagogue having no structural similarities to those of known secretagogues including sulfonylureas. Our data indicate that DSC108 and its sodium salt form (DSC108-Na) have strong insulinotropic effects both in vitro and in vivo and that the pancreatic β-cell K_{ATP} channel is a target of DSC108. In addition, oral administration of DSC108-Na improved glucose tolerance in the Goto-Kakizaki (GK) rat, which is a model of type 2 diabetes with impaired insulin secretion. Thus, DSC108 serves as a lead compound for development of a novel anti-diabetic agent.

Material and Methods

In silico similarity search

Similarity search using two-dimensional (2D) structural fingerprint (TGTFOP) was applied. 2D structures of tolbutamide, chlorpropamide, acetohexamide, glipizide, and glibenclamide were used as queries, and the fingerprints were then calculated by MOE (CCG Inc., Montreal, Canada). Similarity searches were done by Daylight (Daylight Chemical Information Systems, Aliso Viejo, CA) and 38 compounds were retrieved from a commercially available database produced by Namiki Shoji Co., Ltd (Tokyo, Japan). The similarity metrics (Tanimoto coefficient) towards sulfonylureas were calculated for each compound, and all of the structures with similarity coefficient values of less than 0.75 were excluded. In addition, compounds with sulfonylurea structure were manually excluded.

Insulin secretion measurement

MIN6-K8 cells [19] were washed twice and preincubated for 30 min in medium containing 133.4 mM NaCl, 4.7 mM KCl, 1.2 mM KH_2PO_4, 1.2 mM $MgSO_4$, 2.5 mM $CaCl_2$, 5.0 mM

$NaHCO_3$ and 10 mM HEPES (pH 7.4) (KRBH) containing 0.1% BSA with 2.8 mM glucose. After preincubation, the cells were incubated for 30 min in KRBH containing each stimulus. For perfusion experiments of mouse pancreata, overnight (16 hours)-fasted male mice at 16–20 weeks of age were used as previously reported [20]. In the experiments, mouse pancreata were perfused with KRBH buffer containing 2.8 mM glucose in the presence or absence of 10 μM DSC108. Insulin released in the incubation medium or perfusate was measured by insulin assay kits from CIS Bio international (Gif sur Yvette, France).

Chemicals

Derivatives of compound C8 with various chemical modifications including DSC108 and the sodium-salt of DSC108 were synthesized by NARD Institute Ltd. (Kobe, Japan).

Measurement of intracellular Ca^{2+} level ($[Ca^{2+}]i$)

MIN6-K8 cells were loaded with 5 μM Fura2-AM (Dojindo, Kumamoto, Japan) for 20 min at 37°C in KRBH. The cells were stimulated with indicated secretagogues and the intracellular Ca^{2+} level was measured by a dual-excitation wavelength method (340/380 nm) with a fluorometer (Fluoroskan Ascent CF; Labsystems, Helsinki, Finland).

$[^3H]$glibenclamide displacement experiments

MIN6-K8 cells transfected with human SUR1 were incubated with 10 nM $[^3H]$glibenclamide and with different concentrations of DSC108-Na for 30 min in binding buffer. Bound $[^3H]$glibenclamide was separated by rapid vacuum filtration through Whatmann GF/C filters (Whatmann International, Maidstone, U.K.). The filters were washed three times with 4 ml of ice-cold buffer (shown above) and the radioactivity was determined by liquid scintillation counter.

Electrophysiology

Pancreatic β-cells were isolated from C57BL/6 mice by collagenase digestion methods, as previously described [21]. Isolated β-cells were cultured for 24–48 hours before experiments. Membrane potential recordings from β-cells were performed in the current clamp mode of the patch clamp method at room temperature. The composition of the external solution (HEPES-Tyrode solution) was 143 mM NaCl, 5.4 mM KCl, 1.8 mM $CaCl_2$, 0.5 mM $MgCl_2{\cdot}6H_2O$, 0.33 mM NaH_2PO4, 5.5 mM glucose, and 5 mM HEPES- NaOH (pH 7.4) and that of the internal pipette was 110 mM KOH 110 mM L-aspartate, 20 mM KCl, 1 mM $MgCl_2{\cdot}6H_2O$, 0.1 mM EGTA, 1 mM $CaCl_2$, 1 μM ATP-K_2, and 5 mM HEPES-KOH (pH7.4). Effects of DSC108 (30 μM) and glibenclamide (1 μM) on the membrane potentials were then examined.

The β-cell K_{ATP} channels were reconstituted in COS-1 cells transfected with human SUR1 and human Kir6.2 as previously described [6]. The cells were cultured in Dulbecco's modified Eagle's medium (DMEM, Sigma-Aldrich, St. Louis, MO) supplemented with 10% fetal bovine serum for 24–72 hours at 37°C in a humidified CO_2 incubator before the experiments. Effects of DSC108 on the outward current induced by diazoxide (300 μM) (Sigma Chemical, St. Loius, MO) were examined with the whole-cell clamp and compared with those of glibenclamide and gliclazide (Sigma Chemical). The composition of the external solution was the same as that indicated above. The pipette solution contained 107 mM KCl, 2 mM $MgSO_4$, 11 mM EGTA, 1mM $CaCl_2$, 1 μM ATP-K_2, and 11 mM HEPES-KOH (pH 7.2). In the voltage-clamp mode, a ramp-pulse protocol was used to record the quasi-steady-state membrane current at an interval of 20 s. The membrane potential was held at -40 mV and depolarized to +50 mV in 300 ms. It was then repolarized or hyperpolarized to -100 mV in 500 ms, during which time the change

in the membrane current was automatically plotted against the membrane potential. The concentration-inhibitory effect data of the membrane current measured at 0 mV were fitted and IC_{50} values were obtained using Delta Graph 6 (Delta Point, Polaroid Computing, Tokyo).

Animals

Male C57BL/6JJcl mice were purchased from CLEA Japan, Inc. (Tokyo, Japan). Male GK/Slc rats were purchased from Japan SLC, Inc. (Shizuoka, Japan). All animals were maintained under specific pathogen free conditions at 23 ± 2°C and 55 ± 10% relative humidity with a 12-h light-dark cycle, and were provided with water and a commercial diet CE-2 (CLEA Japan, Inc.) at the Animal Facility of Kobe Biotechnology Research and Human Resource Development Center of Kobe University. At the end of the experiments, animals were sacrificed by cervical dislocation or overdose of anesthesia with pentobarbital sodium. All animal experiments were approved by the Committee on Animal Experimentation of Kobe University and Chiba University, and carried out in accordance with the Guidelines for Animal Experimentation at these universities.

Measurement of plasma concentration of DSC108

Male C57BL/6JJcl mice at 16–28 weeks of age were orally administered 30 mg/kg of the compounds and whole blood samples were collected from the tail vein without anesthesia. Plasma was separated by centrifugation for measurement of the plasma concentration. Anti-diabetic agents from mice plasma were extracted by homogenization with extraction solution (Methanol/H_2O/CH_3Cl) containing internal standard (20 μM methionine sulfone) and centrifugation, filtered through a 5-kD filter, and lyophilized. The dried compounds were reconstituted with MilliQ water and subjected to mass spectrometry. DSC108-Na and sulfonylureas were analyzed using a triple quadrupole mass spectrometer (LCMS-8050; Shimadzu Corporation, Kyoto, Japan) coupled with conventional flow liquid chromatography (Nexera UHPLC; Shimadzu Corporation). The LC separation was performed using Discovery HS F5 column (3 μm, 2.1 mm × 150 mm, Sigma-Aldrich) with binary gradient of 0.1% formic acid in water and 0.1% formic acid in acetonitrile.

Oral glucose tolerance test

Male C57BL/6JJcl mice at 16–30 weeks of age were fasted for 16 hours and given DSC108-Na (10–100 mg/kg body weight) at 20 min prior to glucose loading (1.5 g/kg body weight). Male GK/Slc rats (Japan SLC, Inc., Shizuoka, Japan) at 16–30 weeks of age were fasted for 6 hours and given DSC108-Na (100 mg/kg body weight) at 20 min prior to glucose loading (1.5 g/kg body weight). Whole blood samples were collected from the tail vein without anesthesia and blood glucose levels were measured by Antsense III glucose analyzer (Bayer Yakuhin, Osaka, Japan); ELISA system was used for measurement of serum insulin (Morinaga, Tokyo).

Statistical analysis

The results are presented as mean ± SEM. Differences between the groups were analyzed with the Student's *t* test, paired *t* test, or Dunnet's method as indicated in the legend. $P < 0.05$ was regarded as statistically significant.

Results

Identification of insulin secretagogues by in silico screening in combination with insulin secretion measurement

We carried out in silico screening to search for candidate compounds. For this purpose, similarity search using two dimensional structures of sulfonylureas including tolbutamide,

chlorpropamide, acetohexamide, glipizide, and glibenclamide as query compounds was performed against a commercially available compound database containing about 5 million compounds. Following the similarity search, the compounds possessing the sulfonylurea structure were excluded by visual inspection; 38 compounds were finally selected as candidate compounds (S1 Table).

We then investigated whether these compounds could stimulate insulin secretion by using the mouse insulin-secreting cell line MIN6-K8 [19]. MIN6-K8 cells were stimulated by 10 μM of each of the compounds in the presence of 11.2 mM glucose. Of these, four of them, C8, C9, C22, and C25, were selected as hit compounds possessing strong effects on insulin secretion (>2 fold increase vs. vehicle) (Fig 1A and S1 Table).

These four hit compounds were classified into two groups based on their chemical structures (Fig 1B). The first group includes C8, C9, and C22, all of which possess diphenylsemicarbazide; the second group includes C25, which possesses quinolone. We focused on diphenylsemicarbazide as its insulinotropic effect was unknown.

Structure-activity relationship (SAR) of C8-derivatives

For the SAR study of C8, which exhibited the strongest insulinotropic effect, we obtained a total of 69 of its derivatives from a commercial source and by chemical modifications of C8. By comparing their insulinotropic effects, we found that 1,1-diphenylthiosemicarbazide is critical for the activity. In addition, the introduction of a carboxy group on the 4-position of the cyclohexane ring was found to increase the activity. (S1 Fig). Based on the SAR studies of the cyclohexyl ring substitutions, we designed and synthesized the novel compound DSC108 (Fig 2 and S2 Fig). We also synthesized the sodium-salt of DSC108 (referred to as DSC108-Na) (Fig 2 and S2 Fig), which has higher aqueous solubility than DSC108.

Insulinotropic properties of DSC108 in vitro

We examined the properties of DSC108 to stimulate insulin secretion, using MIN6-K8 cells. The DSC108 had a stronger stimulatory effect on insulin secretion at 10 μM, compared to C8, and stimulated insulin secretion significantly even at 3 μM (Fig 3A). Although C8 showed bell-shaped concentration dependency, DSC108 stimulated insulin secretion in a dose-dependent manner (Fig 3A). We also confirmed that DSC108-Na had the same dose-dependency as DSC108 in insulin secretion from MIN6-K8 cells (S3 Fig).

We then analyzed the dynamics of DSC108-induced insulin secretion by perfusion experiment using wild-type mouse pancreata. In the presence of 2.8 mM glucose, 10 μM of DSC108 markedly and transiently stimulated insulin secretion (Fig 3B).

The effect of DSC108 on $[Ca^{2+}]i$ in pancreatic β-cells

To determine whether DSC108-stimulated insulin secretion is associated with a rise in $[Ca^{2+}]i$, we examined the effect of DSC108 on the dynamics of $[Ca^{2+}]$. DSC108 increased $[Ca^{2+}]i$ immediately after stimulation in a dose-dependent manner in a range of 3 to 30 μM (Fig 4). This result suggests that DSC108 stimulates insulin secretion through increasing $[Ca^{2+}]i$ in pancreatic β-cells.

Inhibition of the β-cell K_{ATP} channel by DSC108 through binding to SUR1

Although C8 does not have the sulfonylurea structure, it was originally identified by similarity search using sulfonylurea as query. We therefore explored the possibility that DSC108 might

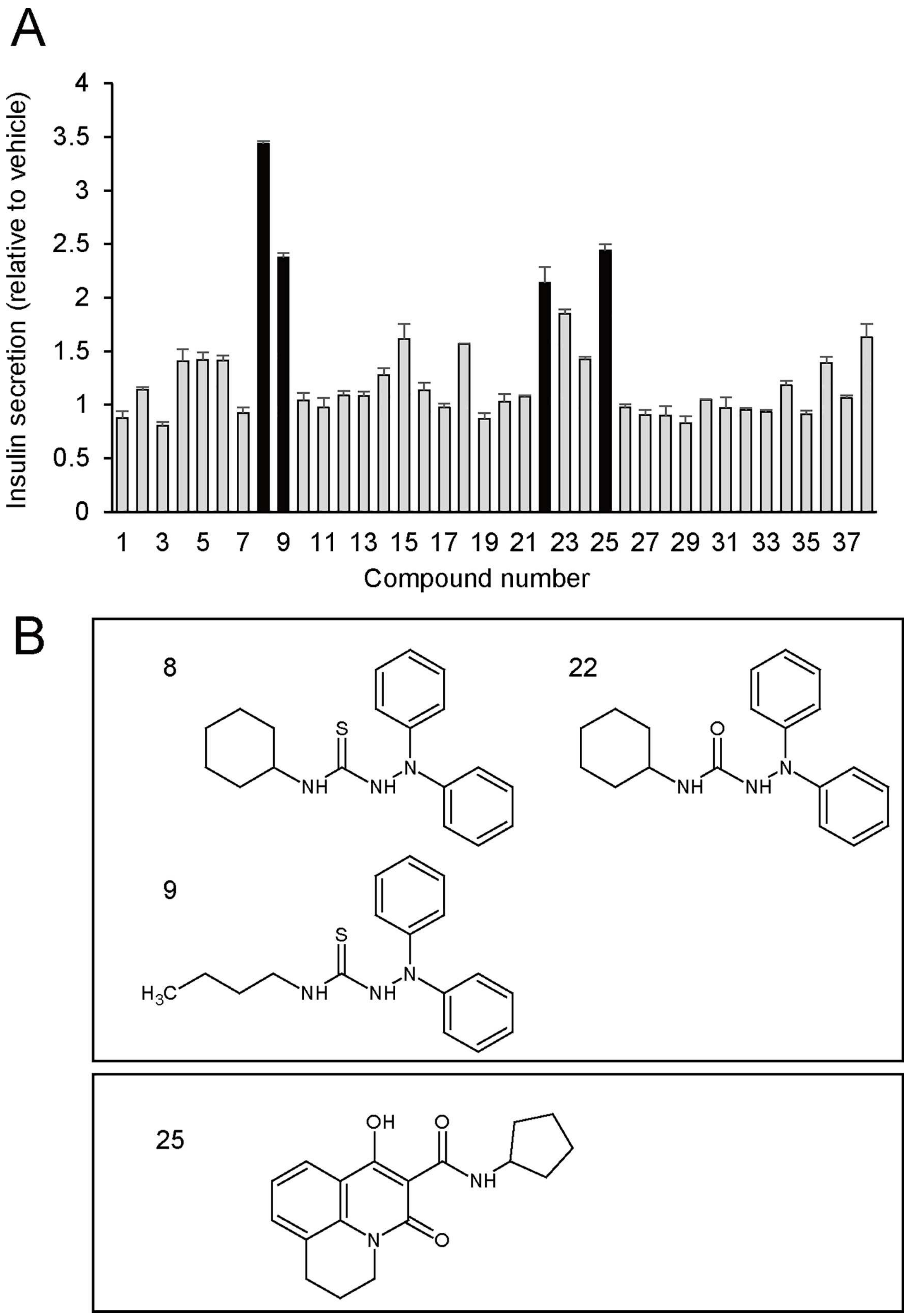
A
Insulin secretion (relative to vehicle)
0
0.5
1
1.5
2
2.5
3
3.5
4
1 3 5 7 9 11 13 15 17 19 21 23 25 27 29 31 33 35 37
Compound number
B
8
S
NH
NH
N
22
O
NH
NH
N
9
S
H_3C
NH
NH
N
25
OH
O
NH
N
O

Fig 1. Insulin secretagogues identified by in silico screening in combination with insulin secretion measurement. (A) Insulin secretion from MIN6-K8 cells stimulated by 10 μM of each compound in the presence of 11.2 mM glucose. Data are fold-increase in insulin secretion relative to vehicle. Values are expressed as mean ± SEM (n = 3 for each compound). (B) Chemical structures of hit compounds. C8, 9, and 22 are diphenylsemicarbazides; C25 is a quinolone.

doi:10.1371/journal.pone.0164785.g001

inhibit the activity of the β-cell K_{ATP} channel. We first examined the binding of DSC108 to the sulfonylurea receptor SUR1, a subunit of the β-cell K_{ATP} channel, in COS-1 cells transfected with human SUR1 [22] using radiolabeled glibenclamide. We utilized DSC108-Na because of

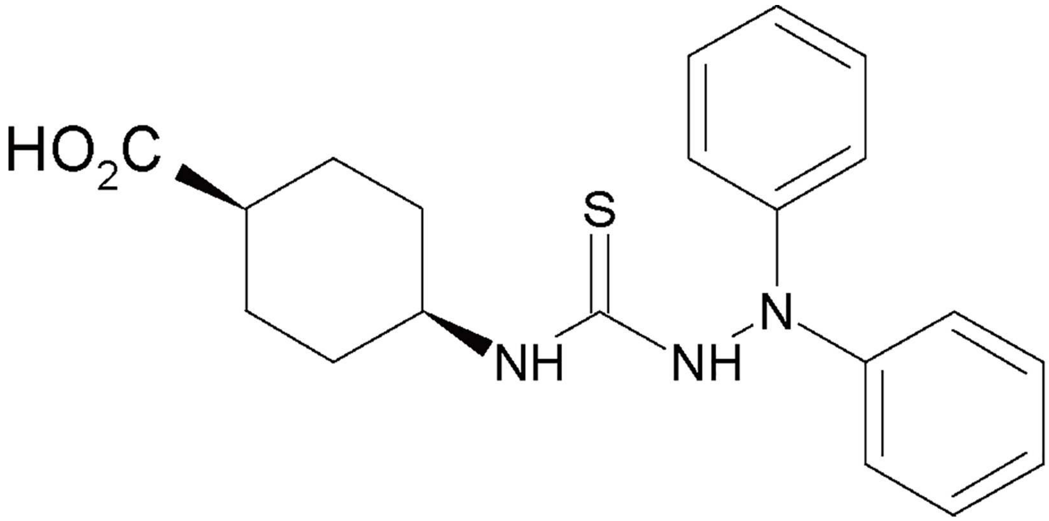

DSC108

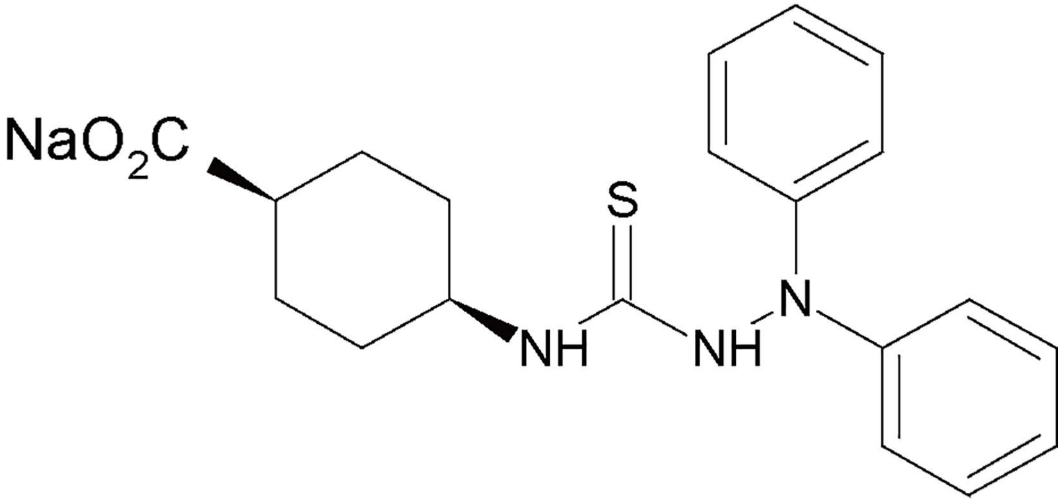

DSC108-Na

Fig 2. Chemical structures of DSC108 and its sodium-salt form (DSC108-Na).

doi:10.1371/journal.pone.0164785.g002

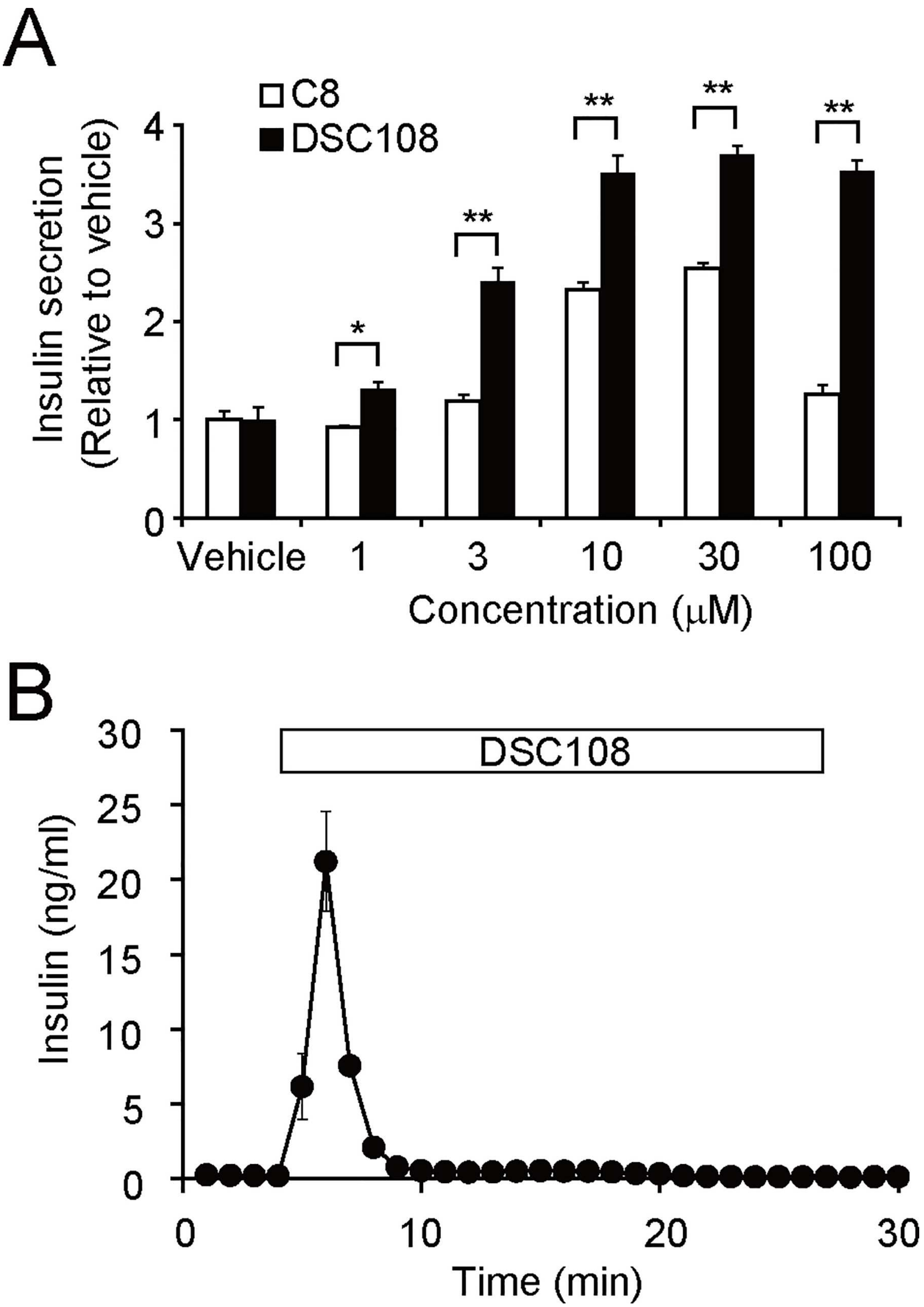
A
C8
DSC108
Insulin secretion
(Relative to vehicle)
4
3
2
1
0
*
**
**
**
**
Vehicle
1
3
10
30
100
Concentration (μM)
B
DSC108
30
25
20
15
10
5
0
Insulin (ng/ml)
0
10
20
30
Time (min)

Fig 3. Insulin secretory properties of DSC108. (A) Effect of C8 and DSC108 on insulin secretion from MIN6-K8 cells. Cells were stimulated by each concentration of compound in the presence of 11.2 mM glucose for 30 min. Data are fold-increase in insulin secretion relative to vehicle. Values are expressed as mean ± SEM (n = 3 for each compound). $^*P < 0.05$, $^{**}P < 0.01$ (Student unpaired *t* test). (B) Effect of DSC108 at 10 μM on the dynamics of insulin secretion from mouse perfused pancreata in the presence of 2.8 mM glucose. Values are expressed as mean ± SEM (n = 3).

doi:10.1371/journal.pone.0164785.g003

its high aqueous solubility. As shown in the displacement curve, 300 μM and 1 mM of DSC108-Na inhibited interaction between ^{3}H-labeled glibenclamide and human SUR1 (Fig 5A), indicating that DSC108 and glibenclamide share the same binding site in SUR1.

To determine whether DSC108 can inhibit activity of the β-cell K_{ATP} channel, we performed electrophysiological experiments. As K_{ATP} channels are important in controlling the membrane potential in pancreatic β-cells, we first examined the effect of DSC108 on membrane potential. DSC108 produced membrane depolarization followed by oscillatory membrane depolarizations in primary cultured pancreatic β-cells, as shown in Fig 6A. DSC108 at a concentration of 30 μM significantly increased the membrane potential from -69 ± 2 mV to -54 ± 4 mV in β-cells (Fig 6B). Glibenclamide (1 μM) also depolarized the membrane from -74 ± 4 mV to -36 ± 8 mV (Fig 6). Since K_{ATP} channels are crucial in determining the membrane potential of pancreatic β-cells, we examined the effect of DSC108 on K_{ATP} channels in COS-1 cells transfected with the human β-cell K_{ATP} channel subunits Kir6.2 and SUR1. Fig 5B shows the representative quasi-steady-state membrane current traces elicited by ramp pulses. DSC108 inhibited the diazoxide-induced K^+ currents in a concentration-dependent manner (IC_{50}, 2.3 μM) as well as those of glibenclamide and gliclazide (Fig 5C). Both DSC108 and glibenclamide inhibited the diazoxide-induced outward current.

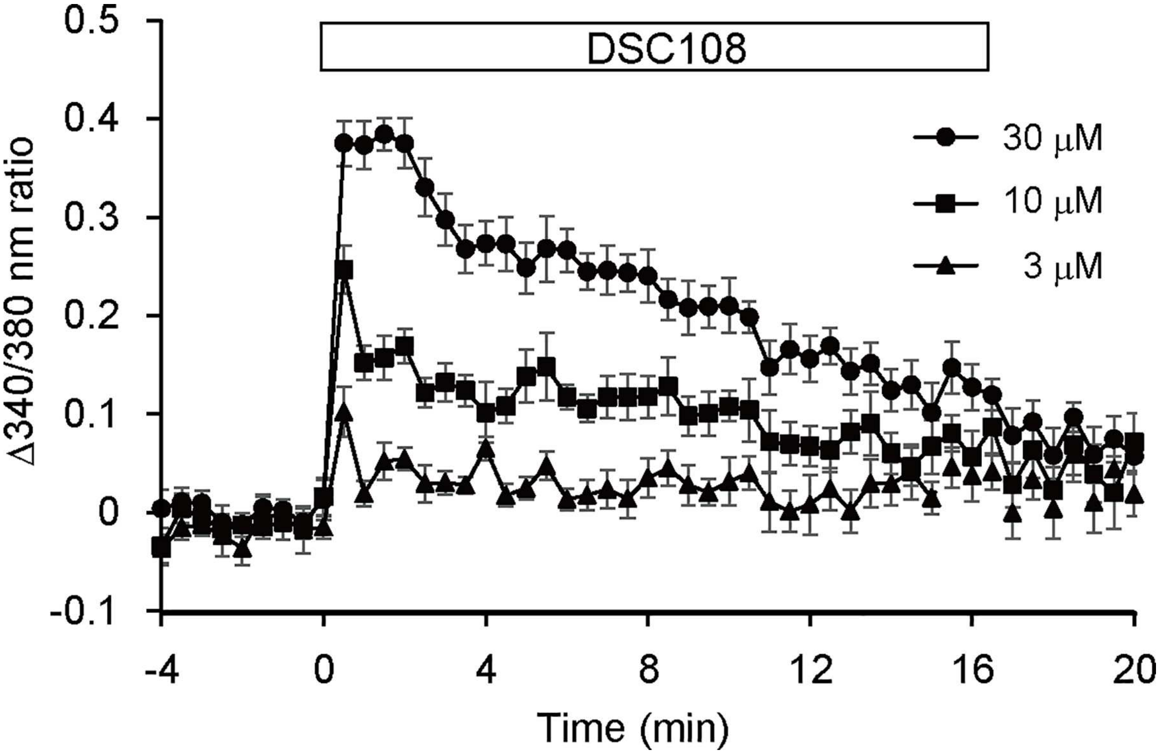

Fig 4. Effect of DSC108 on intracellular Ca^{2+} dynamics in MIN6-K8 cells. Change in intracellular Ca^{2+} concentration induced by DSC108 in the presence of 2.8 mM glucose in MIN6-K8 cells. Data are expressed as change in the ratio of 340nm/380nm fluorescence. Values are expressed as mean ± SEM (n = 4 for each condition).

doi:10.1371/journal.pone.0164785.g004

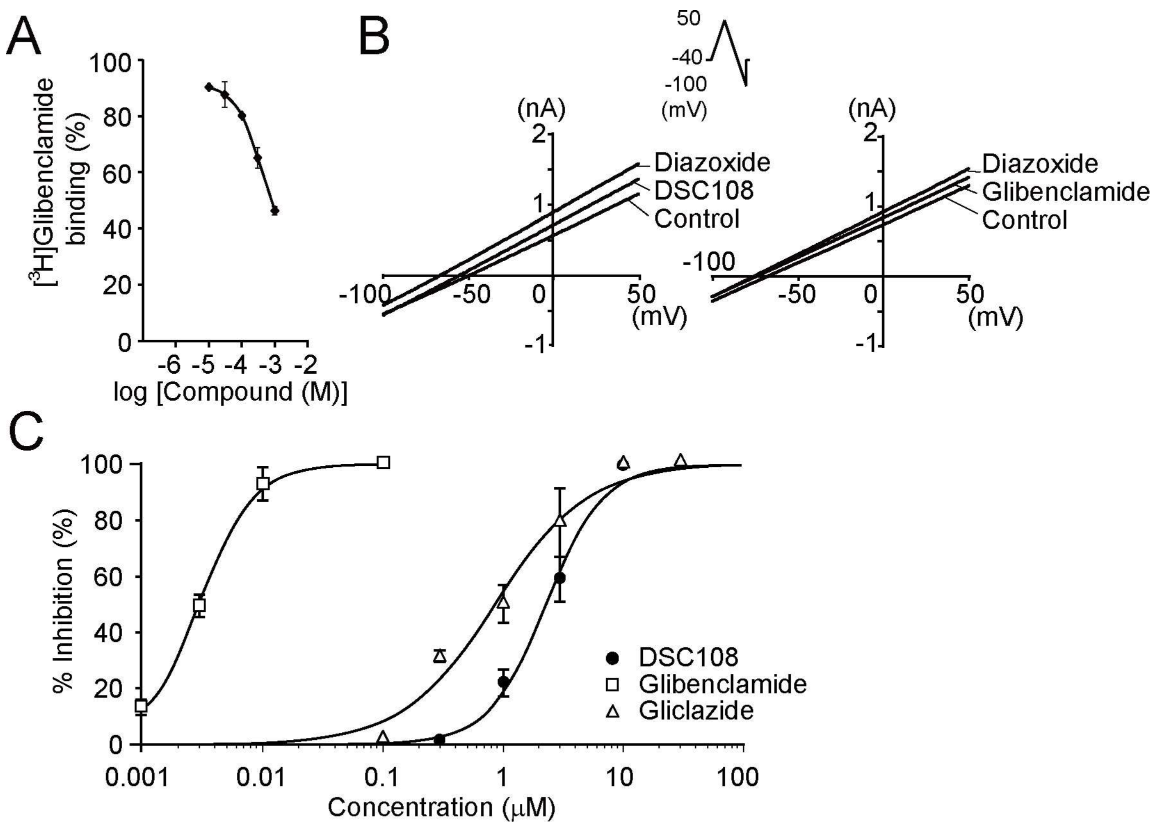

Fig 5. Effects of DSC108 and DSC108-Na on β-cell K_{ATP} channels. (A) Inhibition of [^{3}H]glibenclamide binding to human SUR1 by DSC108-Na. [^{3}H]glibenclamide binding to human SUR1 is displaced by unlabeled DSC108-Na. Values are presented as mean ± SEM (n = 4). (B) Actual current traces recorded from COS-1 cells transfected with human SUR1 and human Kir6.2. The quasi-steady-state membrane current was recorded in the voltage-clamp mode using the ramp-pulse protocol (Inset), and plotted against membrane potential. Both DSC108 (3 μM) and glibenclamide (0.003 μM) inhibited the diazoxide (300 μM)-induced outward current. (C) Concentration-response curves for the inhibitory effects of DSC108, gliclazide, and glibenclamide on diazoxide (300 μM)-induced potassium current at 0 mV. The IC_{50} values for the inhibitory effects of DSC108, gliclazide, and glibenclamide on the diazoxide-induced current are 2.3 μM, 0.86 μM and 0.003 μM, respectively. Each point represents mean ± SEM of 1–6 cells.

doi:10.1371/journal.pone.0164785.g005

These results strongly suggest that DSC108 inhibits the K_{ATP} channels in pancreatic β-cells by binding to SUR1.

Glucose-lowering effect of DSC108 in vivo

As DSC108 stimulates insulin secretion from MIN6-K8 cells and perfused mouse pancreata, we examined whether DSC108 has a glucose-lowering effect in vivo.

To analyze the pharmacokinetic property of DSC108-Na, we first measured its plasma concentrations in the mice by LC-MS. DSC108-Na was orally administered at a dose of 30 mg/kg; its plasma concentration was significantly increased to 72.4 μM at 30 min, followed by a rapid decrease (Fig 7A). In contrast, the plasma concentration of gliclazide, a sulfonylurea used in clinical practice, retained a higher concentration even after 2 hours (Fig 7A). We also found that DSC108-Na (30 mg/kg) significantly increased the plasma insulin level at 20 min after oral administration (Fig 7B). The glucose-lowering effect of DSC108-Na was then evaluated by oral glucose tolerance test in wild-type mice. Glucose (1.5 g/kg) was administered orally 20 min after treatment with DSC108-Na or vehicle (CMC, carboxymethyl cellulose). DSC108-Na dose-dependently suppressed the rises in glucose levels, compared to that in vehicle-treated

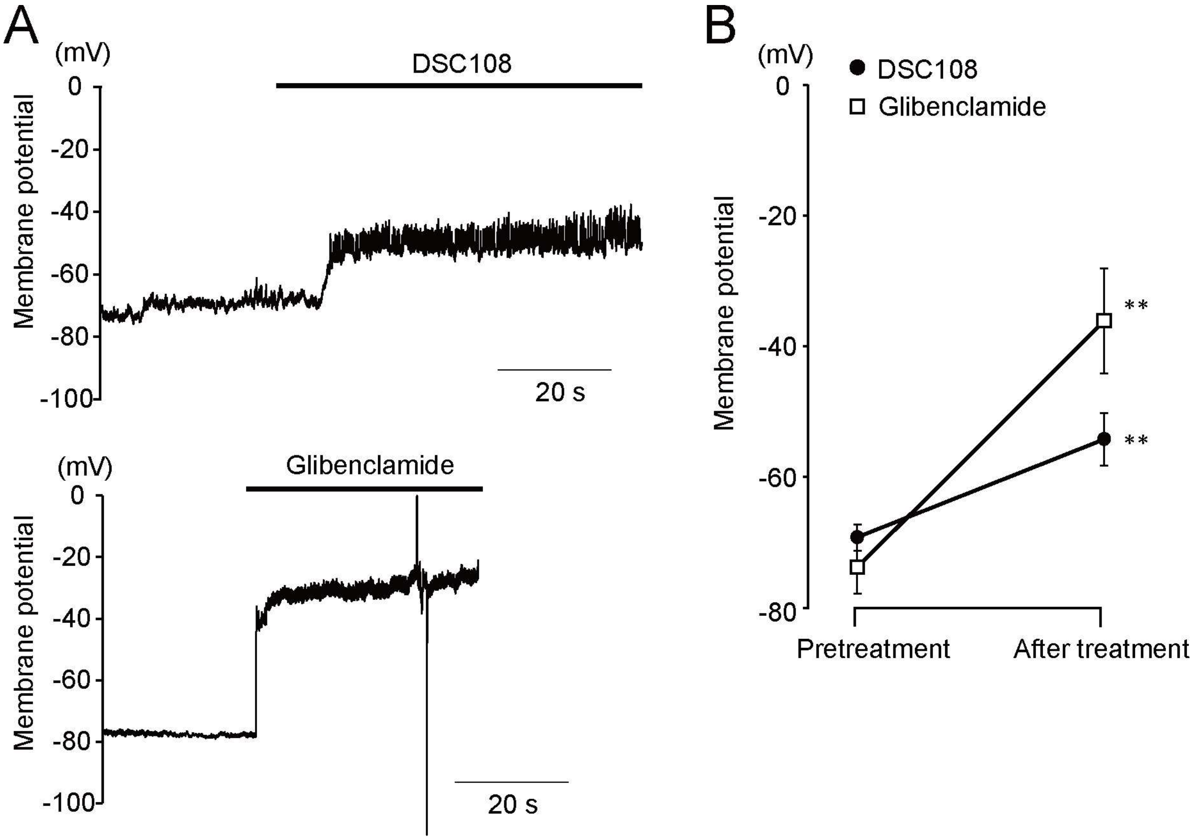

Fig 6. Effects of DSC108 and glibenclamide on the membrane potential of pancreatic β-cells. Membrane potential recording of isolated β-cells was performed by the patch-clamp method in the current clamp mode. (A) Representative membrane potential changes after treatment with 30 μM DSC108 or 1 μM glibenclamide. (B) Summarized data of membrane potential changes after treatment with 30 μM DSC108 (n = 7) or 1 μM glibenclamide (n = 4). Values are expressed as mean ± SEM. **P < 0.01 vs. pretreatment (Student paired *t* test).

doi:10.1371/journal.pone.0164785.g006

mice. In addition, DSC108-Na did not induce hypoglycemia at any time point (Fig 7C). Because aqueous solubility is thought to be an important factor determining its bioavailability, we examined the difference in glucose-lowering effect between DSC108 and DSC108-Na. DSC108-Na, which possesses higher aqueous solubility than DSC108, tended to have a stronger glucose-lowering effect than DSC108 (S4 Fig) despite their exertion of similar insulinotropic effects in vitro (S3 Fig).

Improvement of glucose intolerance in an animal model of type 2 diabetes by DSC108

To determine whether DSC108 has anti-diabetic action, we examined the effect of DSC108-Na on blood glucose levels in the GK rats. The GK rat is a model of non-obese type 2 diabetes with defective insulin secretion associated with impaired glucose metabolism in pancreatic β-cells [23]. We found that oral administration of DSC108-Na (100 mg/kg) significantly increased the plasma insulin level at 20 min after oral administration (Fig 8A). Indeed, in GK rats, blood glucose levels after oral glucose loading were significantly lower in DSC108-pretreated rats than in vehicle-pretreated rats (Fig 8B), indicating that DSC108 has an anti-hyperglycemic effect in type 2 diabetes by stimulating insulin secretion. These results suggest that DSC108 has a potential anti-diabetic effect.

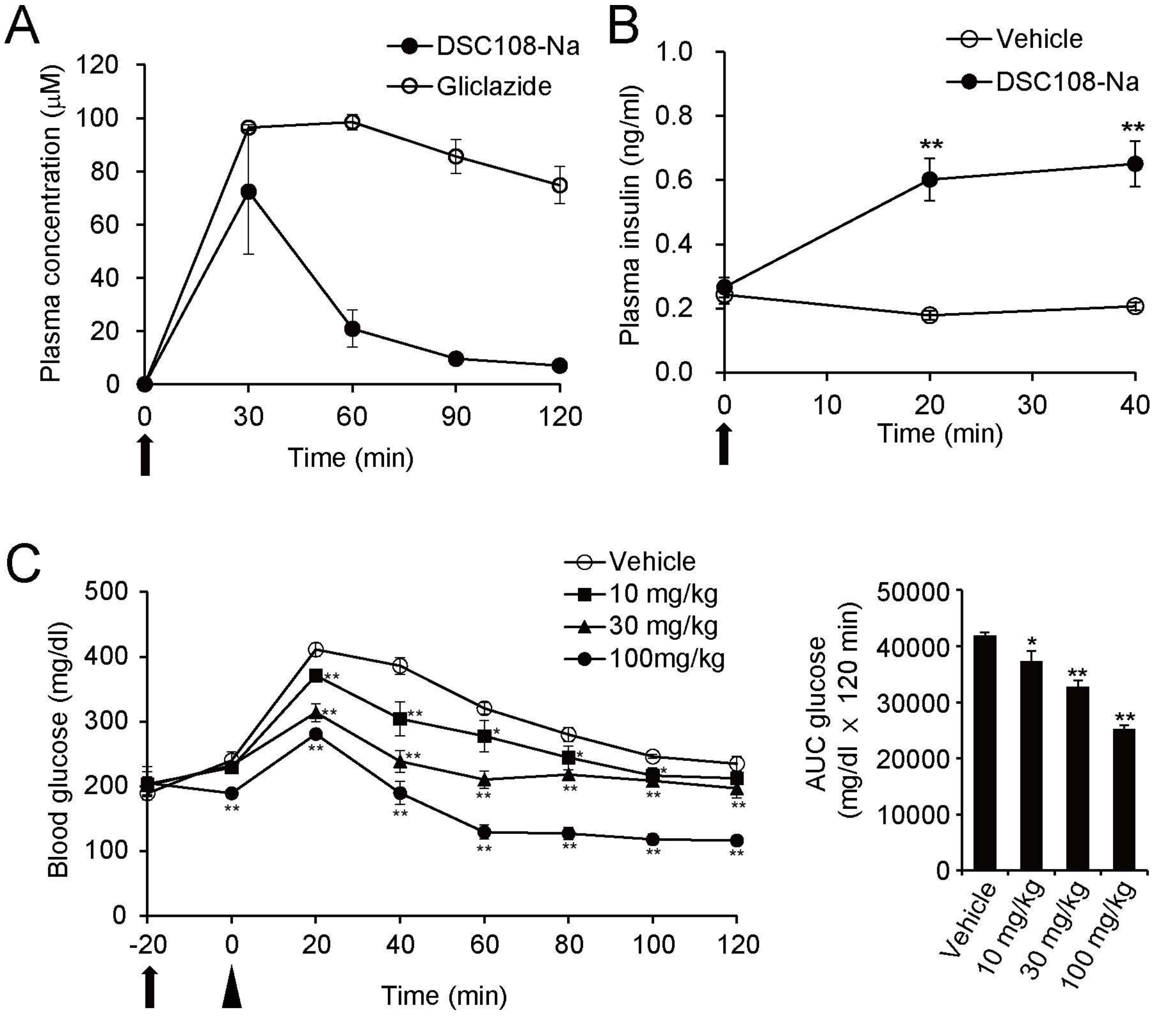

Fig 7. Effects of DSC108-Na in vivo. (A) Plasma concentrations of DSC108-Na and gliclazide. 30 mg/kg of each compound was orally administered to wild type mice (n = 3 for each group) and blood samples were collected every 30 min for 2 hours from the tail vein. Concentration of the compound was analyzed by LC-MS. (B) Insulinotropic effect of DSC108-Na in vivo. 30 mg/kg of DSC108-Na was orally administered to wild type mice and plasma insulin concentrations were measured. Data are expressed as mean ± SEM (n = 4 for each group). **P < 0.01 (Student unpaired *t* test). (C) Glucose-lowering effect of DSC108-Na in OGTT. Changes in blood glucose levels after oral glucose load following administration of DSC108-Na (left). Vehicle or each concentration of DSC108-Na was administered orally at -20 min and glucose (1.5g/kg) was administered orally at 0 min. AUC of glucose is represented in bar graphs (right). Data are expressed as mean ± SEM (n = 4 for each group). Arrow and arrowhead indicate the administration of compound and glucose, respectively. *P < 0.05, **P < 0.01 vs. vehicle group (Dunnet's method).

doi:10.1371/journal.pone.0164785.g007

Discussion

To identify candidate compounds for stimulation of insulin secretion, we used fingerprint-based similarity search by in silico screening. Although high-throughput screening is still the standard method for hit identification, in silico screening is an alternative approach to search for candidate compounds. It is especially useful to evade many cost- and time-consuming processes of drug discovery [24]. In this study, the chemical fingerprints of sulfonylureas were generated with the fingerprints of Typed Graph Triangles Frequency Overlap Planarity (TGTFOP) [25]. By using TGTFOP, followed by exclusion of sulfonylurea-containing compounds by visual inspection, in combination with insulin secretion assay, we attempted to

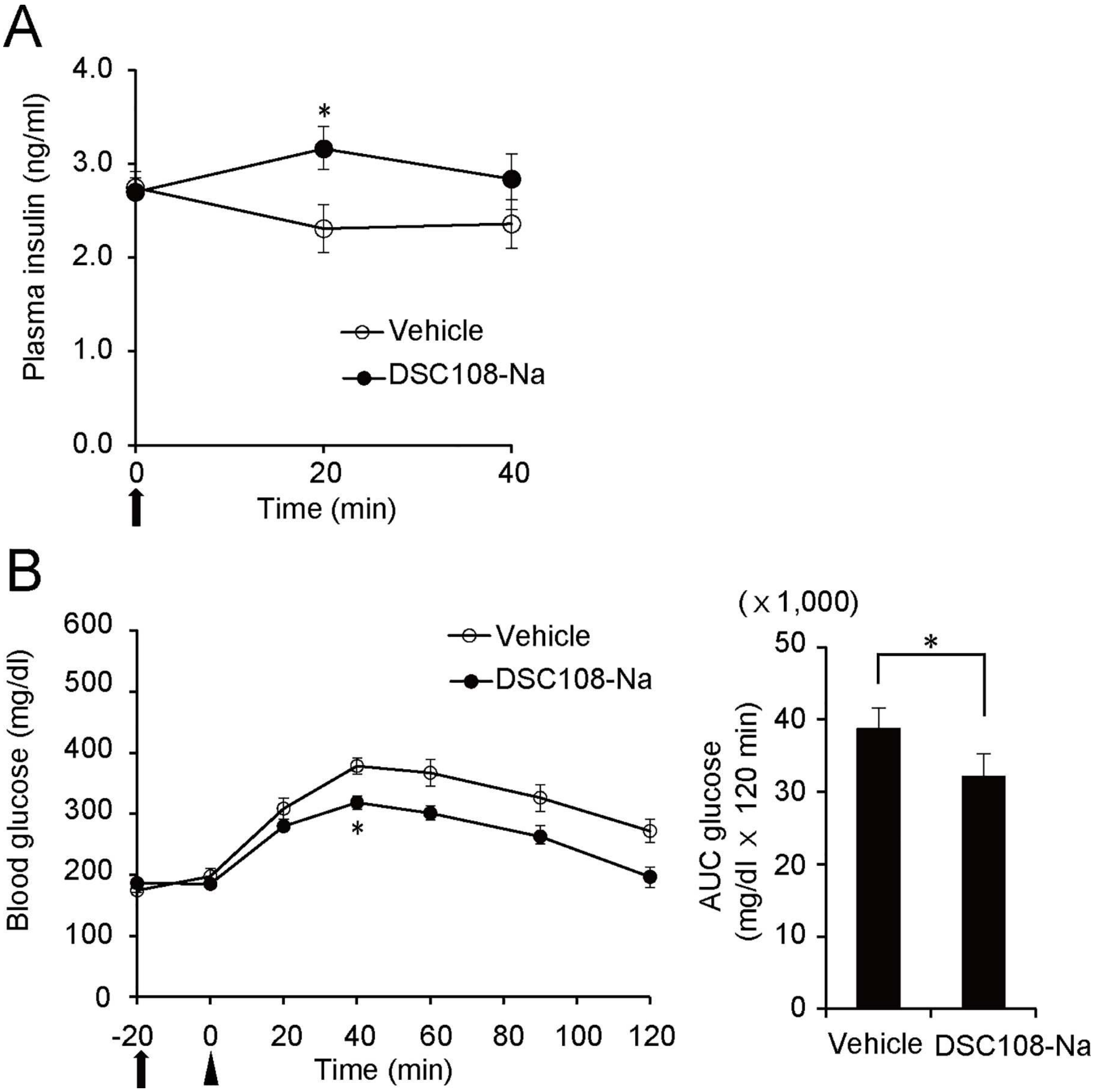

Fig 8. Improvement of glucose tolerance in GK rats by DSC108-Na. (A) Insulinotropic effect of DSC108-Na in vivo. 30 mg/kg of DSC108-Na was orally administered to GK rats and plasma insulin concentrations were measured. Data are expressed as mean ± SEM (n = 6 for each group). *P < 0.05 (paired *t* test). (B) Changes in blood glucose levels after oral glucose load following administration of DSC108-Na in GK rats (left). Vehicle or 100 mg/kg of DSC108-Na was orally administered to rats at -20 min, and glucose was orally loaded at 0 min. AUC of glucose is represented in bar graphs (right). Data are expressed as mean ± SEM (n = 6 for each group). Arrow and arrowhead indicate the administration of compound and glucose, respectively. *P < 0.05 (paired *t* test).

doi:10.1371/journal.pone.0164785.g008

identify novel compounds with an insulinotropic effect. As a result, we discovered a novel diphenylthiosemicarbazide, designated DSC108, which stimulates insulin secretion both in vitro and vivo. DSC108 has no structures common to other insulin secretagogues reported to date.

Intracellular Ca^{2+} measurement, competitive binding inhibition between glibenclamide and DSC108 on SUR1, and electrophysiological study revealed that the β-cell K_{ATP} channel is the primary target of DSC108. Thus, similarly to sulfonylureas and glinides, DSC108 is considered to stimulate insulin secretion by direct inhibition of the K_{ATP} channels in pancreatic β-cells.

It has been shown recently that in addition to inhibition of the K_{ATP} channels, sulfonylureas activate the cAMP-sensor protein Epac2A, an exchange protein activated by cyclic-AMP 2 [11, 12, 26]. We therefore investigated whether DSC108 could activate Epac2A by fluorescence resonance energy transfer (FRET) experiment [11]. DSC108 did not induce FRET change at 10 μM in Epac2A sensor-transfected MIN6-K8 cells (S5 Fig), indicating that DSC108 stimulates insulin secretion in an Epac2A-independent manner.

We also found that DSC108 improves glucose tolerance in the GK rat, a model of type 2 diabetes with impaired insulin secretion [27]. It has been shown that, although ATP production by glucose metabolism is impaired in β-cells of GK rats, the activity of the K_{ATP} channels in the β-cells remains intact [28]. According to our present data in vivo, DSC108 appears to improve glucose tolerance in GK rats by stimulating insulin secretion through inhibition of the K_{ATP} channels.

Hypoglycemia is a non-neglectable side-effect of sulfonylureas, and is primarily caused by its prolonged efficacy [29, 30]. The half-life of sulfonylureas administered in vivo are relatively long in general (3–10 hours), whereas that of DSC108 is less than 30 min (Fig 7A), which is considerably shorter than that of sulfonylureas. The pharmacokinetic property of DSC108 resembles that of glinides, which are known as short-acting insulin secretagogues that target the β-cell K_{ATP} channels [22, 31]. Higher post-prandial glucose levels are a risk factor for the cardiovascular complications of diabetes. Considering that DSC108 improves glucose tolerance in animal models of type 2 diabetes, DSC108 may have a beneficial effect by suppressing the development of this complication. The other characteristic feature of DSC108 is its high aqueous solubility; the aqueous solubility of the sulfonylureas and glinides is generally low [32]. It is thought that the poor aqueous solubility of these drugs leads to slower drug absorption and results in low bioavailability [33]. Solubility of orally administered drugs is an important factor for appropriate pharmacological response [34]. Therefore, improvement of drug solubility remains one of the most important goals in the drug development process, especially for oral-drug delivery systems [35]. DSC108, especially in its sodium salt form, shows a very high aqueous solubility compared to those of sulfonylureas and glinides, which might contribute to higher bioavailability.

It is known that activating mutations of the K_{ATP} channel cause neonatal diabetes in human [36–38]. Many of these patients can be switched from insulin to sulfonylurea for glycemic control. However, developmental delay, epilepsy, and neonatal diabetes (DEND) syndrome, which is a severe form of neonatal diabetes with disorder of the nervous system, are relatively less responsive to sulfonylurea [37]. It will be interesting to learn if DSC108 can pass through the blood brain barrier and block K_{ATP} channels in the brain to improve these neural symptoms.

Although further studies are needed both in vitro and in vivo, DSC108 might well serve as a lead compound in development of a new type of anti-diabetic drug with beneficial properties distinct from those of existing insulin secretagogues. In addition, the approach in the present study affords a useful option for identification of novel insulin secretagogues for treatment of type 2 diabetes.

Supporting Information

S1 Fig. Effects of site-directed carboxylation of C8-derivatives on insulin secretion. (A) Structures of carboxylated C8-derivatives. Carboxylation to various sites on cyclohexane were introduced to C41, one of the C8-derivatives. (B) Effects of carboxylated derivatives on insulin secretion. MIN6-K8 cells were stimulated by 100 μM of each compound. Data are shown as fold-increase in insulin secretion relative to vehicle. Data are expressed as mean ± SEM (n = 3 for each compound)
(TIF)

S2 Fig. Synthesis of DSC108 and DSC108-Na.
(TIF)

S3 Fig. Effects of DSC108 and DSC108-Na on insulin secretion in MIN6-K8 cells. Insulin secretion from MIN6-K8 cells stimulated by 3 or 10 μM of each compound in the presence of 11.2 mM glucose. Data are shown as fold-increase in insulin secretion relative to vehicle. Data are expressed as mean ± SEM (n = 3 for each compound). NS, not significant (Student unpaired *t* test).
(TIF)

S4 Fig. Effects of DSC108 and DSC108-Na in vivo. Blood glucose levels after oral glucose load following administration of DSC108 and DSC108-Na were monitored. Vehicle or 30 mg/kg of DSC108 or DSC108-Na were administered orally at -20 min and glucose (1.5g/kg) was administered orally at 0 min. Data are expressed as mean ± SEM (n = 4 for each group). Arrow and arrowhead indicate the administration of compound and glucose, respectively. $^{**}P < 0.01$ vs. vehicle group (Dunnet's method)
(TIF)

S5 Fig. Effect of DSC108 on Epac2A activation in MIN6-K8 cells. Effect of DSC108 on Epac2A activation in MIN6-K8 cells was assessed by FRET experiment as reported previously [11]. MIN6-K8 cells were transfected with mouse wild-type Epac2A FRET sensor. FRET emission ratio from the cells stimulated by 10 μM of DSC108 or 10 μM 8-pCPT (8-pCPT-2'-*O*-Me-cAMP, an Epac-selective cAMP analog) was monitored as previously described [12].
(TIF)

S1 Table. Characteristics of 38 compounds for the first screening. Structures, values of TGTFOP calculated by in silico similarity search, and activities obtained by the first screening (Fig 1A) are shown.
(TIF)

Acknowledgments

The authors thank N. Inoue and T. Furuya for in silico screening and helpful discussions. The authors are also grateful to R. Hoshikawa for her technical assistance.

Author Contributions

Conceptualization: KS IM SS.

Data curation: KS NY IM KM AM HN.

Formal analysis: KS KH YR NY CS KM AM SS.

Funding acquisition: KS SS.

Investigation: KS KH YR NY CS KM AM.

Methodology: KS NY IM SS.

Project administration: IM NY HN SS.

Resources: AM HN SS.

Supervision: SS.

Validation: KS KH CS AM.

Visualization: KS.

Writing – original draft: KS.

Writing – review & editing: NY HT IM HN SS.

References

1. Stein SA, Lamos EM, Davis SN. A review of the efficacy and safety of oral antidiabetic drugs. Expert Opin Drug Saf. 2013; 12:153–75. doi: 10.1517/14740338.2013.752813 PMID: 23241069
2. Ashcroft FM, Rorsman P. Electrophysiology of the pancreatic beta-cell. Prog Biophys Mol Biol. 1989; 54:87–143. PMID: 2484976
3. Aguilar-Bryan L, Bryan J. Molecular biology of adenosine triphosphate-sensitive potassium channels. Endocr Rev. 1999; 20:101–35. doi: 10.1210/edrv.20.2.0361 PMID: 10204114
4. Henquin JC. Triggering and amplifying pathways of regulation of insulin secretion by glucose. Diabetes. 2000; 49:1751–60. PMID: 11078440
5. Seino S, Miki T. Physiological and pathophysiological roles of ATP-sensitive K+ channels. Prog Biophys Mol Biol. 2003; 81:133–76. PMID: 12565699
6. Inagaki N, Gonoi T, Clement JPt, Namba N, Inazawa J, Gonzalez G, et al. Reconstitution of IKATP: an inward rectifier subunit plus the sulfonylurea receptor. Science. 1995; 270:1166–70. PMID: 7502040
7. Sakura H, Bond C, Warren-Perry M, Horsley S, Kearney L, Tucker S, et al. Characterization and variation of a human inwardly-rectifying-K-channel gene (KCNJ6): a putative ATP-sensitive K-channel subunit. FEBS Lett. 1995; 367:193–7. PMID: 7796919
8. Seino S. ATP-sensitive potassium channels: a model of heteromultimeric potassium channel/receptor assemblies. Annu Rev Physiol. 1999; 61:337–62. doi: 10.1146/annurev.physiol.61.1.337 PMID: 10099692
9. Ashcroft FM, Gribble FM. New windows on the mechanism of action of K(ATP) channel openers. Trends Pharmacol Sci. 2000; 21:439–45. PMID: 11121575
10. Nyenwe EA, Jerkins TW, Umpierrez GE, Kitabchi AE. Management of type 2 diabetes: evolving strategies for the treatment of patients with type 2 diabetes. Metabolism. 2011; 60:1–23. doi: 10.1016/j.metabol.2010.09.010 PMID: 21134520
11. Zhang CL, Katoh M, Shibasaki T, Minami K, Sunaga Y, Takahashi H, et al. The cAMP sensor Epac2 is a direct target of antidiabetic sulfonylurea drugs. Science. 2009; 325:607–10. doi: 10.1126/science.1172256 PMID: 19644119
12. Takahashi T, Shibasaki T, Takahashi H, Sugawara K, Ono A, Inoue N, et al. Antidiabetic sulfonylureas and cAMP cooperatively activate Epac2A. Sci Signal. 2013; 6:ra94. doi: 10.1126/scisignal.2004581 PMID: 24150255
13. Takahashi H, Shibasaki T, Park JH, Hidaka S, Takahashi T, Ono A, et al. Role of Epac2A/Rap1 signaling in interplay between incretin and sulfonylurea in insulin secretion. Diabetes. 2015; 64:1262–72. doi: 10.2337/db14-0576 PMID: 25315008
14. Renstrom E, Barg S, Thevenod F, Rorsman P. Sulfonylurea-mediated stimulation of insulin exocytosis via an ATP-sensitive K+ channel-independent action. Diabetes. 2002; 51 Suppl 1:S33–6. PMID: 11815455
15. Barg S, Renstrom E, Berggren PO, Bertorello A, Bokvist K, Braun M, et al. The stimulatory action of tolbutamide on Ca2+-dependent exocytosis in pancreatic beta cells is mediated by a 65-kDa mdr-like P-glycoprotein. Proc Natl Acad Sci U S A. 1999; 96:5539–44. PMID: 10318919
16. Ozanne SE, Guest PC, Hutton JC, Hales CN. Intracellular localization and molecular heterogeneity of the sulphonylurea receptor in insulin-secreting cells. Diabetologia. 1995; 38:277–82. PMID: 7758873
17. Nagashima K, Takahashi A, Ikeda H, Hamasaki A, Kuwamura N, Yamada Y, et al. Sulfonylurea and non-sulfonylurea hypoglycemic agents: pharmacological properties and tissue selectivity. Diabetes Res Clin Pract. 2004; 66 Suppl 1:S75–8. doi: 10.1016/j.diabres.2003.12.011 PMID: 15563985
18. Drwal MN, Griffith R. Combination of ligand- and structure-based methods in virtual screening. Drug Discov Today Technol. 2013; 10:e395–401. doi: 10.1016/j.ddtec.2013.02.002 PMID: 24050136
19. Iwasaki M, Minami K, Shibasaki T, Miki T, Miyazaki J, Seino S. Establishment of new clonal pancreatic beta-cell lines (MIN6-K) useful for study of incretin/cyclic adenosine monophosphate signaling. J Diabetes Investig. 2010; 1:137–42. doi: 10.1111/j.2040-1124.2010.00026.x PMID: 24843422
20. Miki T, Minami K, Shinozaki H, Matsumura K, Saraya A, Ikeda H, et al. Distinct effects of glucose-dependent insulinotropic polypeptide and glucagon-like peptide-1 on insulin secretion and gut motility. Diabetes. 2005; 54:1056–63. PMID: 15793244

21. Shibasaki T, Takahashi H, Miki T, Sunaga Y, Matsumura K, Yamanaka M, et al. Essential role of Epac2/Rap1 signaling in regulation of insulin granule dynamics by cAMP. Proc Natl Acad Sci U S A. 2007; 104:19333–8. doi: 10.1073/pnas.0707054104 PMID: 18040047

22. Sunaga Y, Gonoi T, Shibasaki T, Ichikawa K, Kusama H, Yano H, et al. The effects of mitiglinide (KAD-1229), a new anti-diabetic drug, on ATP-sensitive K+ channels and insulin secretion: comparison with the sulfonylureas and nateglinide. Eur J Pharmacol. 2001; 431:119–25. PMID: 11716850

23. Ostenson CG, Khan A, Abdel-Halim SM, Guenifi A, Suzuki K, Goto Y, et al. Abnormal insulin secretion and glucose metabolism in pancreatic islets from the spontaneously diabetic GK rat. Diabetologia. 1993; 36:3–8. PMID: 8436249

24. Braga RC, Alves VM, Silva AC, Nascimento MN, Silva FC, Liao LM, et al. Virtual screening strategies in medicinal chemistry: the state of the art and current challenges. Curr Top Med Chem. 2014; 14:1899–912. PMID: 25262801

25. Ewing T, Baber JC, Feher M. Novel 2D fingerprints for ligand-based virtual screening. J Chem Inf Model. 2006; 46:2423–31. doi: 10.1021/ci060155b PMID: 17125184

26. Seino S, Takahashi H, Takahashi T, Shibasaki T. Treating diabetes today: a matter of selectivity of sulphonylureas. Diabetes Obes Metab. 2012; 14 Suppl 1:9–13. doi: 10.1111/j.1463-1326.2011.01507.x PMID: 22118705

27. Goto Y, Kakizaki M, Masaki N. Spontaneous Diabetes Produced by Selective Breeding of Normal Wistar Rats. Proceedings of the Japan Academy. 1975; 51:80–5.

28. Tsuura Y, Ishida H, Okamoto Y, Kato S, Sakamoto K, Horie M, et al. Glucose sensitivity of ATP-sensitive K+ channels is impaired in beta-cells of the GK rat. A new genetic model of NIDDM. Diabetes. 1993; 42:1446–53. PMID: 8375584

29. Ohnota H, Koizumi T, Tsutsumi N, Kobayashi M, Inoue S, Sato F. Novel rapid- and short-acting hypoglycemic agent, a calcium(2s)-2-benzyl-3-(cis-hexahydro-2-isoindolinylcarbonyl) propionate (KAD-1229) that acts on the sulfonylurea receptor: comparison of effects between KAD-1229 and gliclazide. J Pharmacol Exp Ther. 1994; 269:489–95. PMID: 8182516

30. Thule PM, Umpierrez G. Sulfonylureas: a new look at old therapy. Curr Diab Rep. 2014; 14:473. doi: 10.1007/s11892-014-0473-5 PMID: 24563333

31. Mogami H, Shibata H, Nobusawa R, Ohnota H, Satou F, Miyazaki J, et al. Inhibition of ATP-sensitive K + channel by a non-sulfonylurea compound KAD-1229 in a pancreatic beta-cell line, MIN 6 cell. Eur J Pharmacol. 1994; 269:293–8. PMID: 7895769

32. Remko M. Theoretical study of molecular structure, pK(a), lipophilicity, solubility, absorption, and polar surface area of some hypoglycemic agents. Journal of Molecular Structure-Theochem. 2009; 897:73–82.

33. Amidon GL, Lennernas H, Shah VP, Crison JR. A theoretical basis for a biopharmaceutic drug classification: the correlation of in vitro drug product dissolution and in vivo bioavailability. Pharm Res. 1995; 12:413–20. PMID: 7617530

34. Leuner C, Dressman J. Improving drug solubility for oral delivery using solid dispersions. Eur J Pharm Biopharm. 2000; 50:47–60. PMID: 10840192

35. Blickle JF. Meglitinide analogues: a review of clinical data focused on recent trials. Diabetes Metab. 2006; 32:113–20. PMID: 16735959

36. Ashcroft FM. ATP-sensitive potassium channelopathies: focus on insulin secretion. J Clin Invest. 2005; 115:2047–58. doi: 10.1172/JCI25495 PMID: 16075046

37. Pearson ER, Flechtner I, Njolstad PR, Malecki MT, Flanagan SE, Larkin B, et al. Switching from insulin to oral sulfonylureas in patients with diabetes due to Kir6.2 mutations. N Engl J Med. 2006; 355:467–77. doi: 10.1056/NEJMoa061759 PMID: 16885550

38. Remedi MS, Nichols CG. Hyperinsulinism and diabetes: genetic dissection of beta cell metabolism-excitation coupling in mice. Cell Metab. 2009; 10:442–53. doi: 10.1016/j.cmet.2009.10.011 PMID: 19945402
